# Complete Genome Sequence of *Escherichia coli* Strain WG5

**DOI:** 10.1128/genomeA.01403-17

**Published:** 2018-01-11

**Authors:** Lejla Imamovic, Maria-Anna Misiakou, Eric van der Helm, Gianni Panagiotou, Maite Muniesa, Morten Otto Alexander Sommer

**Affiliations:** a Novo Nordisk Foundation Center for Biosustainability, Technical University of Denmark, Lyngby, Denmark; b Leibniz Institute for Natural Product Research and Infection Biology, Hans Knöll Institute, Jena, Germany; c Department of Genetics, Microbiology and Statistics, Microbiology Section, Faculty of Biology, University of Barcelona, Barcelona, Spain

## Abstract

*Escherichia coli* strain WG5 is a widely used host for phage detection, including somatic coliphages employed as standard ISO method 10705-1 (2000). Here, we present the complete genome sequence of a commercial *E. coli* WG5 strain.

## GENOME ANNOUNCEMENT

*Escherichia coli* is a common commensal of the human and animal gut. *E. coli* strain WG5 is used as a host to detect somatic coliphages, proposed to be an indicator of human and animal fecal contamination of water, sediments, and sludge, as described in ISO method 10705-1 (2000) (1). This is an easily applicable and affordable method for water quality and contamination management in water treatment facilities (2). Other studies employ a similar method using strain WG5 as a host to detect temperate infectious phages from food (3, 4). The applicability of the method is based on the high sensitivity of the *E. coli* WG5 host to infection by somatic coliphages (5). *E. coli* WG5 (6) is a nalidixic acid-resistant mutant of *E. coli* C, also known as strain CN, and is publicly available in the ATCC (ATCC number 700078). *E. coli* strain WG5 possesses an attenuated host restriction-modification system and contains only the core part of the lipopolysaccharide (LPS), increasing susceptibility to phage infections (5, 7).

Bacterial DNA was isolated using a DNeasy blood and tissue kit (Qiagen), according to the manufacturer’s protocols. DNA libraries were prepared using a TruSeq Nano kit (Illumina) and sequenced on a MiSeq platform (2 × 300 bp). In parallel, genomic DNA was used to prepare barcoded DNA with Native barcoding kit 1D (product number EXP-NBD103; Oxford Nanopore Technologies), together with Ligation sequencing kit 1D (SQK-LSK108; Oxford Nanopore Technologies). DNA was sequenced using R9.4 chemistry (FLO-MIN106; Oxford Nanopore Technologies), and the raw signal was base called using Albacore 1.2.6. The sequences were assembled *de novo* into a single contig using the Unicycler hybrid assembler (8), with default settings.

The WG5 strain has a circular complete genome of 4,592,887 bp. Genome annotation was acquired from NCBI Prokaryotic Genome Annotation Pipeline (9), which revealed 4,657 genes, 4,536 coding sequences, 22 rRNAs (5S, 16S, and 23S), 87 tRNAs, and 22 noncoding RNAs. Multilocus sequence types were identified using MSTL (10). No horizontally identified antibiotic resistance genes were detected by using ResFinder (11). The PHASTER prophage finder (12) identified three prophage regions, with two incomplete prophage regions and one questionable prophage region.

### Accession number(s).

The genome sequences have been deposited in GenBank under the accession number CP024090. The version described in this paper is the first version.
